# Learning to encode cellular responses to systematic perturbations with deep generative models

**DOI:** 10.1038/s41540-020-00158-2

**Published:** 2020-11-06

**Authors:** Yifan Xue, Michael Q. Ding, Xinghua Lu

**Affiliations:** 1 Department of Biomedical Informatics, School of Medicine, University of Pittsburgh, Pittsburgh, PA 15206 USA; 2 Department of Pharmaceutical Sciences, School of Pharmacy, University of Pittsburgh, Pittsburgh, PA 15206 USA

**Keywords:** Computational biology and bioinformatics, Computer modelling

## Abstract

Cellular signaling systems play a vital role in maintaining homeostasis when a cell is exposed to different perturbations. Components of the systems are organized as hierarchical networks, and perturbing different components often leads to transcriptomic profiles that exhibit compositional statistical patterns. Mining such patterns to investigate how cellular signals are encoded is an important problem in systems biology, where artificial intelligence techniques can be of great assistance. Here, we investigated the capability of deep generative models (DGMs) to modeling signaling systems and learn representations of cellular states underlying transcriptomic responses to diverse perturbations. Specifically, we show that the variational autoencoder and the supervised vector-quantized variational autoencoder can accurately regenerate gene expression data in response to perturbagen treatments. The models can learn representations that reveal the relationships between different classes of perturbagens and enable mappings between drugs and their target genes. In summary, DGMs can adequately learn and depict how cellular signals are encoded. The resulting representations have broad applications, demonstrating the power of artificial intelligence in systems biology and precision medicine.

## Introduction

A cellular signaling system is a signal processing machine that detects changes in the internal or external environment, encodes these changes as cellular signals, and eventually transmits these signals to effectors, which adjusts cellular responses accordingly. Cellular responses to perturbations often involve changes in transcriptomic programs^1–3^. The investigation of cellular signaling systems is an important task in the field of systems biology. A common approach is to systematically perturb a cellular system with genetic or pharmacological perturbagens and monitor transcriptomic changes in order to reverse engineer the system and gain insights into how cellular signals are encoded and transmitted. This approach has been employed in many large-scale systems biology studies, e.g., the yeast deletion library^4^, the Connectivity Map project^5,6^, and most recently, the Library of Integrated Network-based Cellular Signatures (LINCS)^7,8^. The LINCS project is arguably the most comprehensive systematic perturbation dataset currently available, in which multiple cell lines were treated with over tens of thousands perturbagens (e.g., small molecules or single gene knockdowns), followed by monitoring gene expression profiles using a new technology known as the L1000 assay, which utilizes ~1000 (978) landmark genes to infer the entire transcriptome^7^.

While there are numerous studies using LINCS data to investigate the mechanism-of-action (MOA) of drugs and to promote clinical translation of MOA information^5,7,9–14^, few studies aim to use the data for learning to represent the cellular signaling system as an information encoder. This would enable examination of how different perturbagens affect the cellular signaling system. It can be imagined that when signaling components at different levels of a signaling cascade are perturbed, the resulting expression data would present compositional statistical structures that can be hard to reverse-engineer. For instance, perturbing an upstream signaling molecule will likely subsume the effect of perturbing its downstream molecules. Capturing such a compositional statistical structure requires models that are capable of representing hierarchical relationships among signaling components. In this study, we developed deep generative models (DGMs) to understand how perturbagens affect the cellular signal encoding process and lead to changes in the gene expression profile.

DGMs are a family of deep learning models that employ a set of hierarchically organized latent variables to learn the joint distribution of a set of observed variables. After training, DGMs are capable of generating simulated data that preserve the same compositional statistical structure as the training data. The hierarchical organization of latent variables is particularly suitable for representing cellular signaling cascades and detecting compositional statistical patterns derived from perturbing different components of cellular systems. The capability to “generate” samples similar to the training data are of particular interest. If a model can accurately regenerate transcriptomic data produced under different perturbations, the model should have learned a representation of the cellular signaling system that enables it to encode responses to perturbations. Such representations could shed light on the MOAs through which perturbagens impact different cellular processes.

**Fig. 1:**
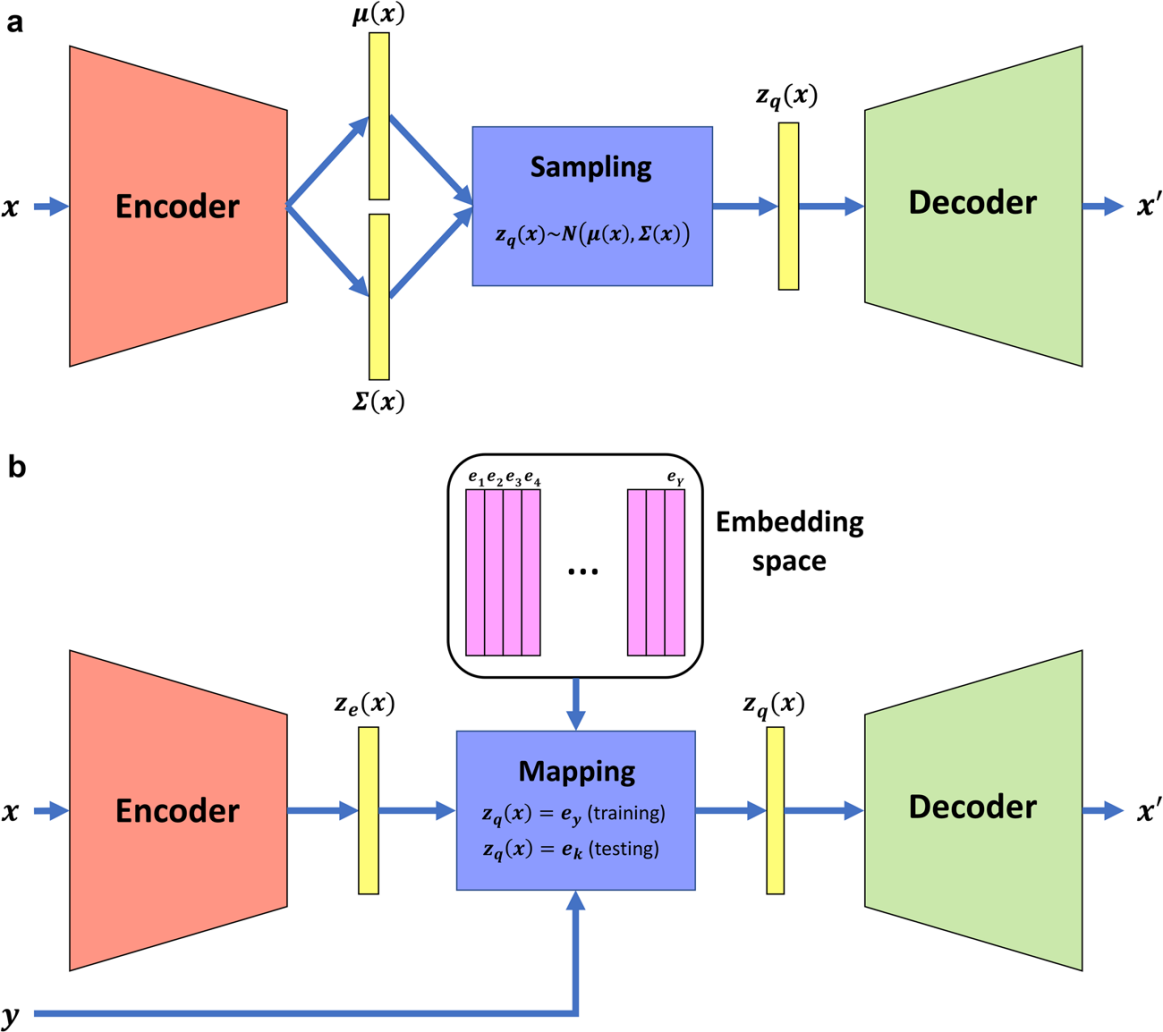
The VAE model and S-VQ-VAE model. **a** The architecture of VAE. The encoder and decoder are two sub-neural networks. An input case is transformed into a mean vector ***μ***(***x***) and a covariance vector ${\boldsymbol{{\Sigma} }}({\boldsymbol{x}})$ by the encoder, from which the encoding vector ${\boldsymbol{z}}_{\boldsymbol{q}}({\boldsymbol{x}})$ is sampled and fed to the decoder to reconstruct the input case. The distribution of the encoding vector is trained to follow a prior standard normal distribution. **b** The architecture of S-VQ-VAE. S-VQ-VAE is an extension of VQ-VAE where the training of the embedding space is guided by the label of the input data. Similar to VAE, an input case is first transformed into an encoding vector ${\boldsymbol{z}}_{\boldsymbol{e}}\left( {\boldsymbol{x}} \right)$ by the encoder. During training, the encoding vector is replaced by the embedding vector ${\boldsymbol{e}}_{\boldsymbol{y}}$ designated to represent the label ${\boldsymbol{y}}$ of data to reconstruct the input case. The embedding vector is updated according to the reconstruction error. During testing, the encoding vector is replaced by the nearest neighbor embedding vector ${\boldsymbol{e}}_{\boldsymbol{k}}$.

In this study, we investigate the utility of two DGMs, the variational autoencoder (VAE) (Fig. 1a)^15–17^ and a new model, the supervised vector-quantized variational autoencoder (S-VQ-VAE) (Fig. 1b), for learning the signal encoding process of cells. We show that the VAEs can reconstruct the LINCS data accurately and also generate new data that are indistinguishable from real observed data. We demonstrate that by adding a supervised learning component to vector-quantized VAE (VQ-VAE)^18^, we are able to summarize the common features of a family of drugs into a single embedding vector and use these vectors to reveal relationships between different families of drugs. Our study represents the pioneering efforts that systematically investigate the power of DGMs for learning how cellular signals are encoded in response to perturbations. Our findings support the use of DGMs as a powerful tool in modeling cell signaling systems.

## Results

### Modeling cellular transcriptomic processes with VAE

We carried out a series of model comparison experiments and selected a deep learning architecture based on model complexity, reconstruction error, and other aspects of performance (see “Methods”). The input and output layers contained 978 nodes, each corresponding to one of the 978 landmark genes in an L1000 expression profile. The internal architecture is composed of three hidden layers in its encoder, with 1000, 1000, and 100 hidden nodes, respectively (Supplementary Fig. 1); the decoder has a reverse architecture as the encoder.

We trained three VAE models independently on datasets consisting of different combinations of samples treated with two types of perturbagens. The first model was trained on the small-molecule perturbagen (SMP) dataset, which contains 85,183 expression profiles from seven cell lines treated with small molecules (Supplementary Table 1). The second model was trained on the genetic perturbagen (GP) dataset, which contains 116,782 expression profiles from nine cell lines with a single gene knockdown (Supplementary Table 1). The third model was trained on the combined SMP and GP dataset (SMGP). We excluded 4649 samples treated with two proteasome inhibitors (bortezomib and MG-132) from the SMGP dataset as these samples form a unique outlier distribution on the principal component analysis (PCA) plot (Supplementary Fig. 2, see Methods). Supplementary Table 2 shows the performance of the three VAE models trained independently on the SMP, GP, and SMGP datasets.

**Fig. 2:**
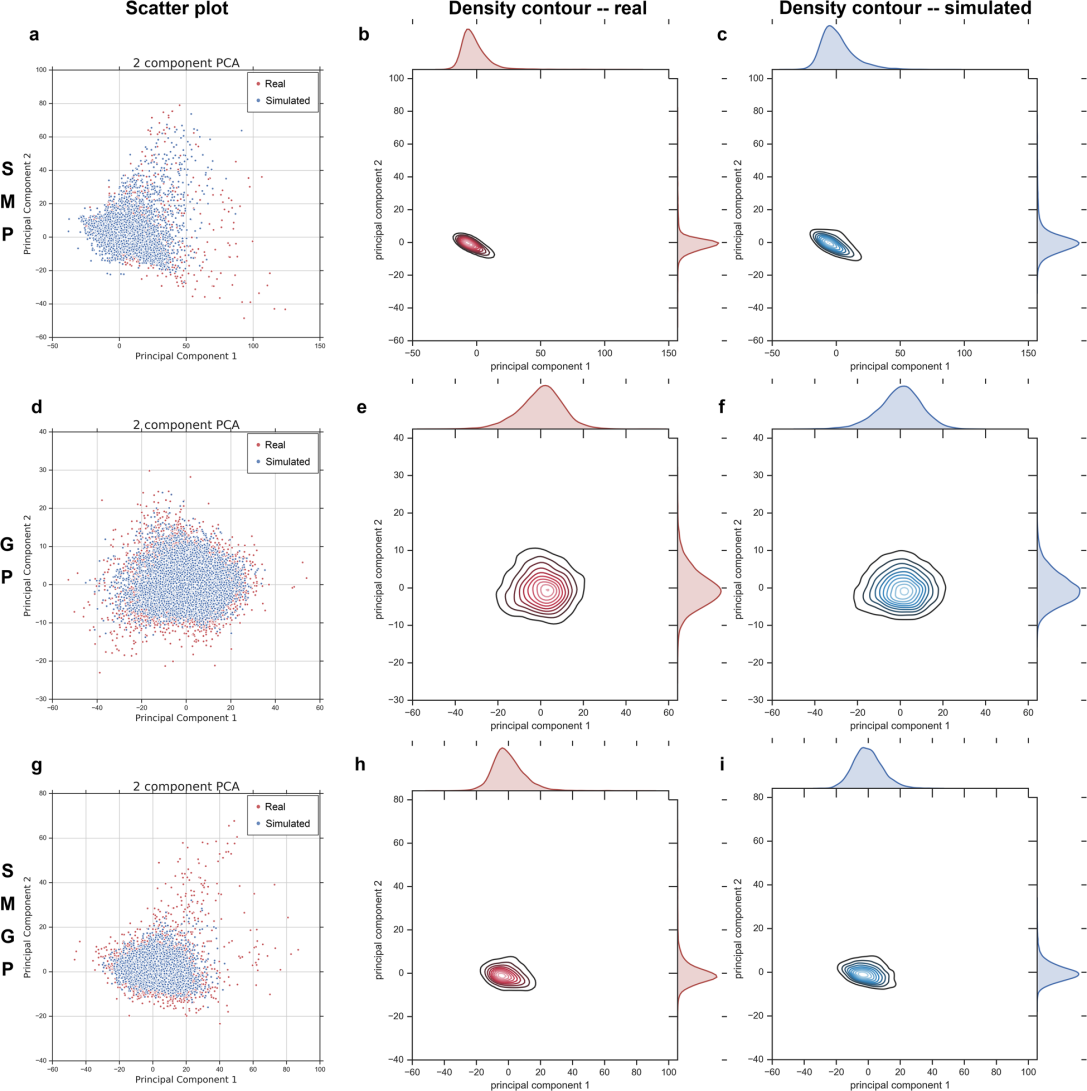
Simulated data of VAE vs. original input data. **a** Scatter plot of simulated data (blue points) generated by SMP-trained VAE and the original SMP data (red points) in the space of the first two PCA components. **b** The density contour of the real data in (**a**). **c** The density contour of the simulated data in (**a**). **d** Scatter plot of simulated data (blue points) generated by GP-trained VAE and the original GP data (red points) in the space of the first two PCA components. **e** The density contour of the real data in (**d**). **f** The density contour of the simulated data in (**d**). **g** Scatter plot of simulated data (blue points) generated by SMGP-trained VAE and the original SMGP data (red points) in the space of the first two PCA components. **h** The density contour of the real data in (**g**). **i** The density contour of the simulated data in (**g**).

To examine whether the trained VAE models learned the distribution of the input data, we generated new data using the trained VAE models and compared their distribution with that of the original input data. For each of the three models, we randomly generated 10,000 samples and projected them along with 10,000 randomly selected original training samples into the first two principal components space (Fig. 2). From the scatter plots in Fig. 2 (a, d, and g), we can see that the VAE-generated data points form a similar distribution in the PCA plot as the input data for all three experiments. The consistency in the centroid location, shape, and range of the density contour indicates that the VAE models are able to capture the major statistical characteristics of the input data distribution (Fig. 2).

We then performed hierarchical clustering analyses to confirm whether the newly generated data are indistinguishable from real data. Using 2000 randomly generated samples and 2000 randomly selected original samples, we conducted hierarchical clustering with 1-Pearson correlation as the distance metric (Supplementary Fig. 3). We cut the dendrogram at 10 clusters and computed a mixing score (see “Methods”) to examine whether the generated data and original data were similarly distributed across clusters. For binary-categorical data, a mixing score is of range [0.5, 1], which gives the average proportion of data from the dominant category in each cluster. A mixing score of 0.5 indicates an even mixture of the two categories of data within all clusters, and a score of 1 indicates a clear separation between the two categories across clusters. For each of the three VAE models, this process of sample generation, selection, and mixing score computation was repeated 50 times. The mean mixing score was 0.594 for SMP-trained VAE with a 95% confidence interval (CI) of [0.589, 0.598], 0.586 for GP-trained VAE with a CI of [0.581, 0.591], and 0.603 for SMGP-trained VAE with a CI of [0.598, 0.608]. These mixing scores indicate that neither real data nor simulated data exhibit dominance in individual hierarchical clusters. Therefore, the generated data cannot be separated from the real data via hierarchical clustering.

### A few signature nodes encode the primary characteristics of an expression profile

To gain a better understanding of how VAEs encode the distribution of diverse input data, we next examined the activation patterns of hidden nodes on different layers of the SMGP-trained VAE model. We paid particular attention to the top hidden layer of 100 nodes that serves as an “information bottleneck” for compressing the original data, because this layer also serves as the starting point for the generation of new samples.

**Fig. 3:**
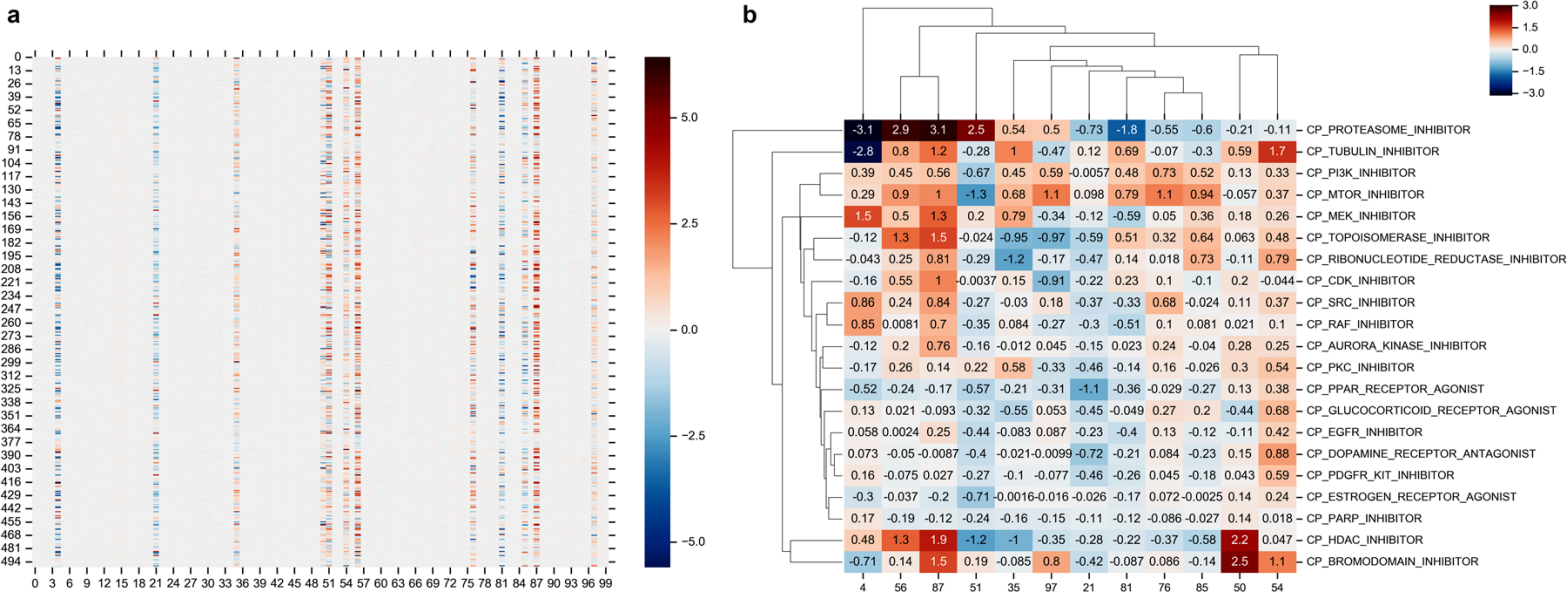
Signature nodes on the top hidden layer of SMGP-trained VAE. **a** The heatmap of the 100 hidden nodes of the top hidden layer for 500 random selected SMC samples. The pseudo-colors represent the values of elements in the encoding vectors. **b** The average of signature nodes for samples treated with major PCLs.

For this analysis, we utilized a subset of the SMP dataset where the samples were treated with small molecules that had been classified into one of the perturbagen classes (PCLs) based on MOA, gene targets, and pathway annotations as defined by the LINCS project^7^. We call this subset the SMP dataset with Class information (SMC) dataset, and it consists of 12,079 samples treated with small molecules from 75 PCLs. For each of these samples, we computed an encoding vector by feeding the expression profile through the encoder of the SMGP-trained VAE to the top hidden layer ($z_q\left( x \right)$ in Fig. 1a). We found that 12 out of 100 nodes in the encoding vector had a high variance in activation values across samples (Fig. 3a). The average values of these nodes show a clear bimodal distribution with one mode formed by the 12 nodes and one mode formed by the others (Supplementary Fig. 4). For the SMC dataset, the average absolute value of these 12 nodes is 0.885 vs. 0.048 of the other hidden nodes, (two-sided t-test *p*-value < 1e−10). Across the SMGP training dataset as a whole, the average absolute value of the 12 nodes is 0.503 and vs. 0.044 of the other nodes (*p* < 1e−10).

For an ordinary VAE model, the prior distribution of the encoding vector is a standard normal distribution with a mean vector $\mu \left( x \right) = 0$ and a diagonal covariance matrix ${\Sigma} \left( x \right) = {\mathrm{diag}}(1)$ (Fig. 1a). During training, an element of the vector shrinks towards 0 unless it is driven by data to deviate from 0. Therefore, the significantly high absolute values taken by these 12 hidden nodes suggest that they encode major signals of input data. From this point forward, we refer to these 12 hidden nodes as the signature nodes.

We investigated whether the patterns of these 12 signature nodes reflect the MOA of drugs by examining their association with PCLs. A PCL was considered a major perturbagen class if at least 150 samples were treated with perturbagens of that class in the LINCS data. Using this definition, there are 21 major PCLs. For each major PCL, we fed the samples treated with perturbagens of the class through the trained VAE encoder and took the average signature node values across samples as a vector representation of the PCL. As shown in Fig. 3b, different PCLs presented different patterns in the signature nodes.

We further examined whether the representations of each PCL revealed similarities between PCLs via hierarchical clustering analysis (Fig. 3b). PCLs that are closely clustered tend to share similar MOAs (Fig. 3b). For example, the mTOR inhibitor and PI3K inhibitor were grouped together according to their consistent activation directions (positive vs. negative) for most signature nodes, and they are both known to impact the PI3K/AKT signaling pathway^19^, where mTOR is a downstream effector of PI3K. Other examples include the grouping of Src inhibitor and Raf inhibitor, where Src is known to activate Ras-c, which in turn activates Raf in the Raf-MEK-ERK kinase cascade^20^; the grouping of topoisomerase inhibitor and ribonucleotide reductase inhibitor, which both impact DNA replication; and the grouping of Aurora kinase inhibitor and PKC inhibitor, where Aurora kinases are essential in mediating the PKC-MAPK signal to the NF-κB/AP-1 pathway^21^. These observations support the idea that the 12 signature nodes preserve crucial information representing an expression profile resulting from a small-molecule perturbation of the cellular system.

**Fig. 4:**
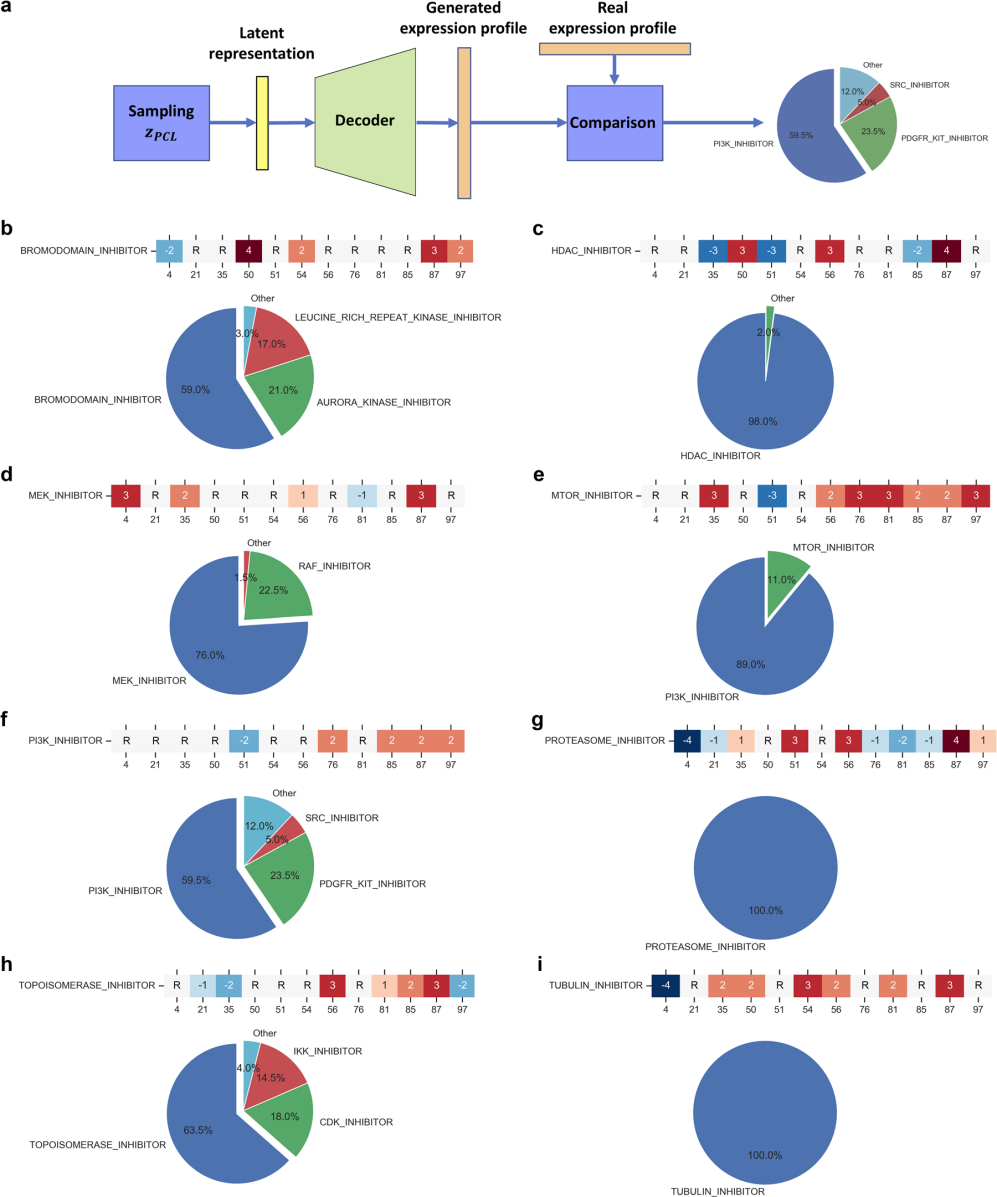
Comparison of data generated based on the signature pattern of PCLs with real data. **a** Diagram illustrating the procedure for generating new data from the signature pattern of a PCL. First, an encoding vector is initialized where the signature nodes are set according to the signature pattern of real samples from the given PCL; the non-signature nodes are randomly initialized by sampling from a standard normal distribution. The vector is then fed through the decoder of the SMGP-trained VAE, and a new expression profile is generated. The new data are compared to real data by computing the nearest neighbor based on Euclidean distance, to see if the new data are closely related to real data of the given PCL. **b**–**i** The composition of real data nearest neighbors of new data generated from latent representations simulating different PCLs. “R” indicates the value of the signature node is not specified but random initialized as non-signature nodes.

To further demonstrate that the primary characteristics of an expression profile are encoded in the 12 signature nodes, we generated new expression profiles to simulate samples treated with a target PCL by manipulating values of the signature nodes to mimic the patterns found the previous experiment. We preset the signature nodes to values similar to the average values of training samples treated with the target PCL as shown in Fig. 3b and randomly initialized the other hidden nodes from a standard normal distribution. In this manner, we randomly generated 500 new samples using the VAE decoder for eight major PCLs. We then compared the randomly generated samples against real samples to see whether their nearest neighbors were from the target PCL (Fig. 4a). The signature node patterns used to generate samples and the similarity of these samples to real samples and associated PCLs are shown in Fig. 4b–i.

In most cases, more than half of the generated data had a nearest neighbor from the target PCLs. For proteasome inhibitor and tubulin inhibitor specifically (Fig. 4g and i), 100% of the generated data were nearest neighbors of real samples from the target PCL, which was repeatedly observed across independent runs. These results agrees with the PCL clustering outcomes in Fig. 3b, where proteasome inhibitor and tubulin inhibitor were found as outliers from the other PCLs with their distinct signature node patterns.

We also noted that the specific value of each signature node did not matter as long as the value correctly reflects the direction, i.e., positive or negative, of the node for a given PCL. This suggests that the major characteristics of a PCL can potentially be encoded into only 12 bits of information. The only pattern that did not have over half of the generated samples mapped to the target PCL was the mTOR inhibitor (Fig. 4e). Most of the samples generated using mTOR signature nodes were closest neighbors of PI3K inhibitor-treated samples. This is reasonable, as mTOR inhibitors act downstream on the same pathway as PI3K inhibitors. For this reason, the former’s effects can be in many cases replicated by the latter. This observation also supports the conclusion that each signature node pattern reflects a specific cellular signaling process, which, after decoding, generates an expression profile that may reflects how the signaling is perturbed.

### Learning global representations of PCLs with S-VQ-VAE

The signature node representations of PCLs discussed above were obtained by averaging over samples treated with small-molecule perturbagens of a PCL. In order to learn a unique, stable global representation for each PCL, we designed another DGM, the S-VQ-VAE, which utilizes the PCL class labels of perturbagens to partially supervise the training process. S-VQ-VAE was extended from VQ-VAE by utilizing the vector-quantized (VQ) technique to discretize the encoding vector space into multiple mutually exclusive subspaces represented by a limited number of embedding vectors and projecting data from each class into its pre-assigned subspace (Fig. 1b, see “Methods”). After training, each embedding vector learns to summarize the global characteristics of a class of data. In this study, we used S-VQ-VAE to learn an embedding vector with a dimension of 1000 for representing each of the 75 PCLs in the SMC dataset (Supplementary Table 2).

**Fig. 5:**
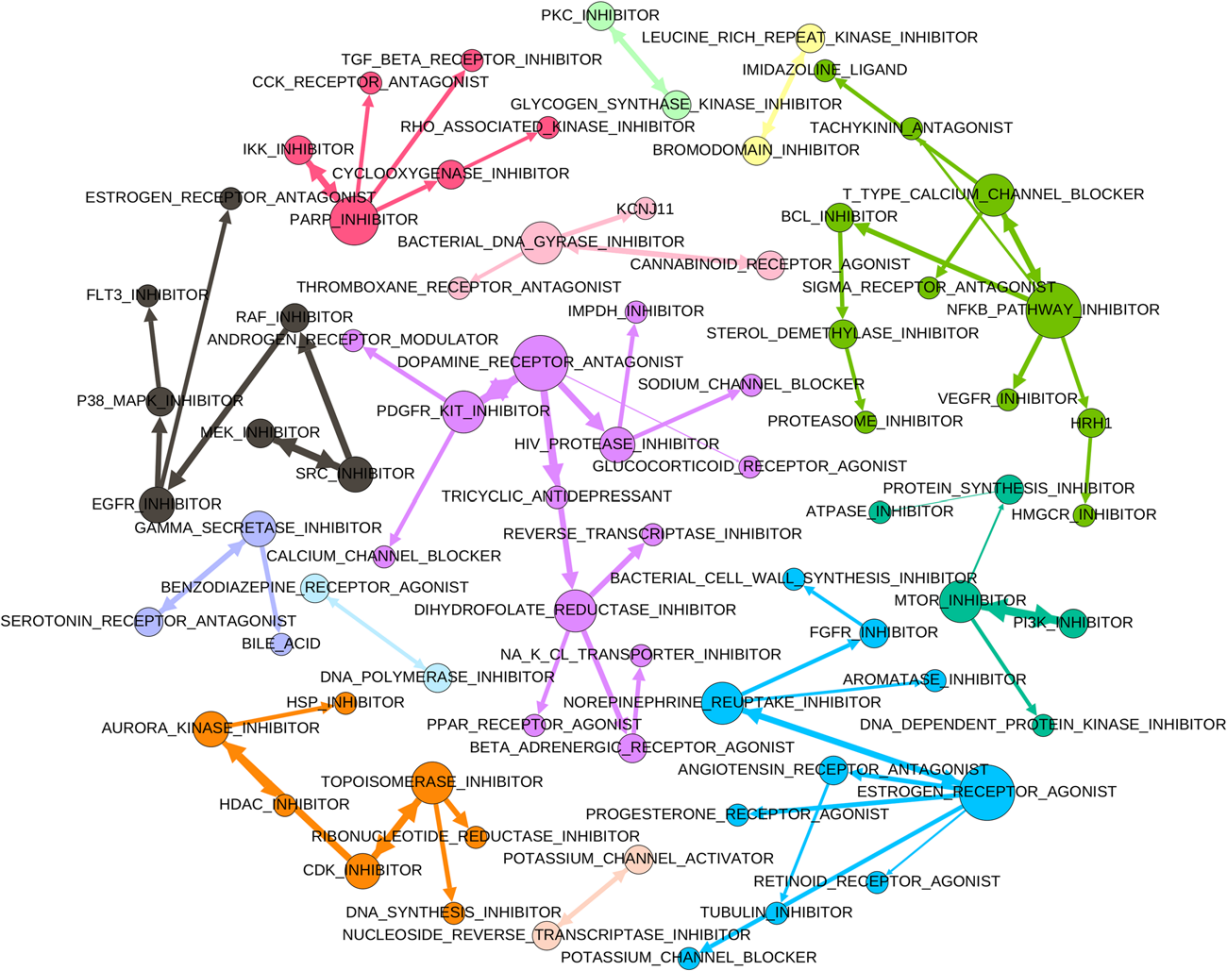
Similarities between PCLs revealed by global PCL representations learned by S-VQ-VAE. A directed edge in the graph indicates that the source node is the nearest node to the target code based on the Pearson correlation between the corresponding representations. The node size is proportional to the out-degree. The edge width is proportional to the correlation. The color of a node indicates the community the node belongs to.

We utilized the embedding vectors to reveal similarities and potential functional relationships between PCLs by comparing each PCL to all the others to identify its nearest neighbor based on Pearson correlation. The nearest neighbor relationships between PCLs are visualized as a directed graph (Fig. 5), in which a directed edge indicates that the source node is the nearest partner to the target node. We also applied the Louvain algorithm^22^ to detect communities (or clusters) among PCLs, and members of different communities are indicated as pseudo-colors (Fig. 5). The modularity score of the communities is 0.875, which indicates significantly denser connections existing between members within communities compared to a randomly assigned network of the same set of PCLs.

Some strong relationships, like bi-directional connections are observed (Fig. 5), and many such relationships correspond to well-documented shared MOAs between the drugs in the connected PCLs. These include the relationships that have also been revealed with the signature node representations above, e.g., the functional similarity between mTOR inhibitors and PI3K inhibitors^19^, and the relationship between MEK inhibitors, Src inhibitors, and Raf inhibitors^20^. Other strong connections were observed between CDK inhibitors and topoisomerase inhibitors, which may reflect coordinated response to mitosis inhibition and DNA damage induction^23,24^, between Aurora kinase inhibitors and HDAC inhibitors which both impact the histone deacetylase pathway^25^, and between gamma-secretase inhibitors, serotonin receptor antagonists, and bile acid that affect amyloid precursor protein processing and lipid metabolism^26,27^.

The members of a PCL community also shed light on the high-level functional theme of the community. For example, the black community on the left of Fig. 5 with Raf, Src, MEK, and EGFR related PCLs may represent drug effects transmitted through the EGFR-RAS-RAF-MEK signaling cascade. The orange community (bottom left of Fig. 5), consisting of inhibitors of Aurora kinase, HDAC, CDK, topoisomerase, ribonucleotide reductase, and DNA synthesis, may represent the signaling transduction for regulating DNA duplication and mitosis. The blue community (bottom right of Fig. 5), with estrogen, progesterone, norepinephrine, and angiotensin may represent the comprehensive effects of perturbing hormones. These findings indicate that the global representations learned with S-VQ-VAE preserve crucial information that reveals the functional impact of different PCLs.

### The VAE latent representations preserve PCL-related information

The latent variables at different levels of the hierarchy of a DGM may encode cellular signals with different degrees of complexity and abstraction^28^. Therefore, we next investigated the information preserved in the latent variables of different hidden layers of the SMGP-trained VAE. To do this, we first represented the SMC samples with seven types of representations, including the raw expression profiles, the latent representations obtained from the five hidden layers of the VAE (across the encoder and decoder), and the 12 signature node values (see “Methods”). We then used these representations to predict the PCL label of the small molecule used to treat each sample by training two multi-classification models, the logistic regression (LR) and the support vector machine (SVM). As shown in Supplementary Table 3, the highest test prediction accuracy was achieved by using the raw expression profiles as input data for both LR and SVM (accuracy 0.5922 and 0.5273 respectively). This was followed by the latent representations of samples extracted from the first hidden layer of the VAE encoder (accuracy 0.5096 for LR and 0.4528 for SVM). The lowest accuracy was obtained using the 12 signature node values as input data (0.3814 for LR and 0.3615 for SVM). Nonetheless, the highest test accuracy achieved with latent representation, 0.5096, was nearly 10 times higher than guessing at random from the 75 unevenly distributed PCLs, 0.0543. These results indicate that although there was information loss with respect to the classification task as the representations become more abstract with deeper hidden layers, the latent representations preserved significant information from the original input data.

### The VAE latent representations enhance drug-target identification

**Fig. 6:**
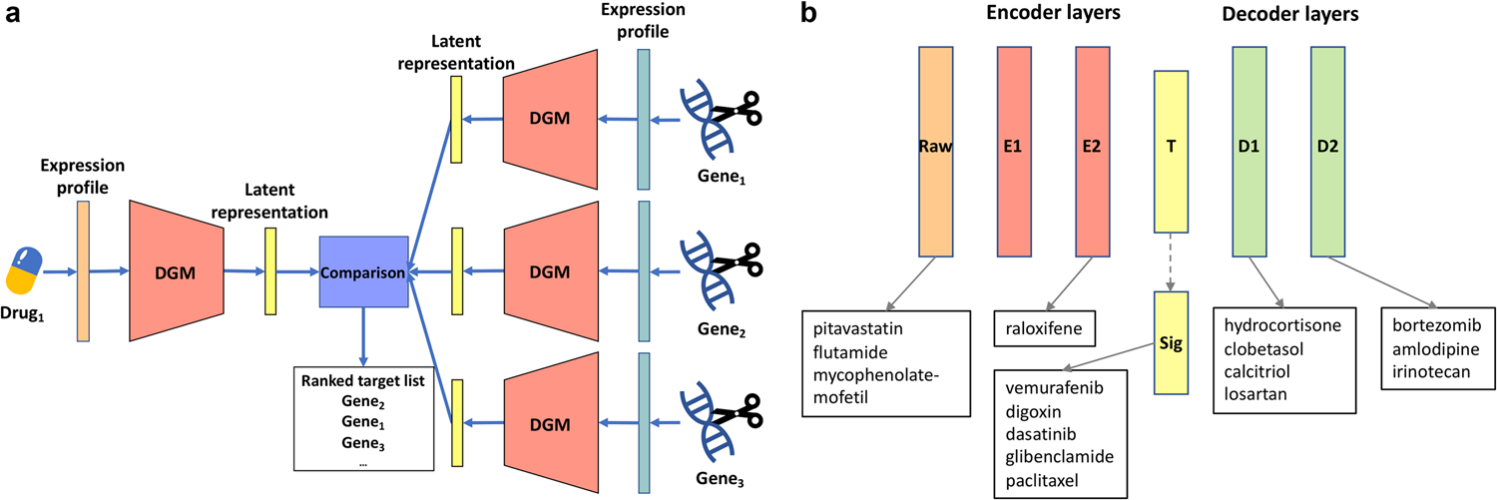
Drug-target prediction with different sample representations for 16 FDA-approved drugs. **a** Diagram illustrating the approach for drug-target prediction. For a given drug, samples treated with the drug are fed to the SMGP-trained VAE to obtain latent representations from different layers of the encoder and decoder. All GP samples are also fed to the VAE to obtain corresponding latent representations and compared with the SMP samples by computing the Pearson correlation. For a given type of representation, genes are ranked according to the correlations with respect to the representations of SMP samples treated with the drug, and the ranks of the top known target of the drug are recorded. **b** The representation type that achieved the best matching (lowest mean rank) of the top known target gene for each drug.

Combining SMP and GP data can help establish connections between the MOAs of small molecules and genetic perturbations, which further help reveal the targets of small molecules^6,12^. A simple approach is to examine whether a pair of perturbagens (a small molecule and a genetic perturbation) leads to similar transcriptomic profiles, or more intriguingly, similar latent representations that reflect the state of the cellular system. Given a known pair of a drug and its target protein, we assumed that treatment with the drug and knockdown of the gene of the protein would result in a similar transcriptomic response reflected in the raw expression profile or VAE-derived latent representations. Based on this assumption, we extracted 16 FDA-approved drugs and their gene targets from ChEMBL database that are also available in LINCS data^12,29^. We computed the Pearson correlations between the representations (either the raw expression profile or a latent representation) of each SMP sample treated by a drug and the corresponding representations of all GP samples. The GP samples and their knockdown genes were then ranked according to the correlations to obtain the ranks of known target genes, in a manner similar to an information retrieval task (Fig. 6a).

We compared different types of representations to identify which were more effective in assigning a higher rank to target genes. As shown in Supplementary Table 4 and Table 1, for different drugs, different representations achieved the best target-retrieval performance as reflected by top rank and mean rank. This suggests that VAEs can encode the impact of different drugs within different layers in the hierarchy that potentially reflect the relative level of drug-target interactions in the cellular signaling network. Figure 6b summarizes the mean rank results where each drug is assigned to the representation layer that produced the best mean rank of its top known target. Most drugs have their best performance achieved with VAE-learned latent representations rather than the raw expression profiles, and for five drugs, the best performance was achieved with the 12-signature-node-representation. Table 1 gives the best rank of the top known target for each drug, which is comparable to Table 1 from Pabon et al.^12^. Even though our approach is essentially an unsupervised learning method based purely on expression data, 13 out of 16 drugs received an equal or better rank than from the previous state of the art random forest model trained with a combination of expression and protein–protein interaction features^12^ (bolded in Table 1).

**Table 1:** The rank of the top known target for 16 FDA-approved drugs from drug-target prediction with different types of representations.

Drug	Target	Raw	E1	E2	T	Sig.	D1	D2
**Pitavastatin**^b^	HMGCR	2^a^	1	1	1	1	2	1
**Bortezomib**	PSMB10, PSMA3, PSMA1, PSMA5, PSMB7, PSMB5, PSMA8, PSMB1	1	1	1	1	1	1	1
**Hydrocortisone**	NR3C1	72	35	37	12	11	3	1
**Vemurafenib**	BRAF	1	1	1	1	1	1	1
**Flutamide**	AR	36	14	39	78	160	29	163
**Clobetasol**	NR3C1	415	10	2	7	16	13	4
**Digoxin**	ATP1A3, FXYD2, ATP1B1	71	2	9	30	15	23	11
**Mycophenolate-Mofetil**	IMPDH2	12	342	31	28	21	18	44
**Dasatinib**	LCK, YES1	25	2	5	5	7	3	2
**Amlodipine**	CACNA1D	62	243	134	111	116	97	58
Calcitriol	VDR	917	164	341	661	211	1018	335
Glibenclamide	KCNJ11	275	497	336	579	307	482	536
**Paclitaxel**	TUBB6, TUBA1A, TUBB2A, TUBB2C	14	71	49	20	18	92	98
Losartan	AGTR1	238	190	370	53	115	253	57
**Irinotecan**	TOP1	308	653	331	18	13	326	504
**Raloxifene**	ESR2	114	115	53	139	72	105	65

*E* encoder layer, *T* top hidden layer, *Sig* signature nodes, *D* decoder layer.

^a^The value for a given drug and a type of representation is the top rank of the drug-target gene(s) among all genes in GP dataset when retrieved and ranked according to the similarity between the representations of SMP samples treated with the drug and representations of all GP samples. A lower rank is better.

^b^Drugs with the lowest rank equal to or lower than the rank reported by Pabon et al. are bolded.

In addition to comparing a SMP sample to all GP samples, we also created two smaller datasets to only include SMP and GP samples that are correlated with the 16 FDA-approved drugs and their targets from ChEMBL and 810 drugs and their targets from the Drug Gene Interaction database (DGIdb)^30^, respectively. For each drug-perturbed sample, we ranked their target genes using the same approach as shown in Fig. 6a and computed average top n recall and proportion of samples with at least one true target retrieved in top n ranked genes for all types of representations (Supplementary Table 5). For both datasets, the best aggregation performance was achieved with latent representations rather than the raw expression profile, which also supports that latent representations are better at revealing drug-target relations.

## Discussion

In this study, we examined the utility of DGMs, specifically VAE and S-VQ-VAE, for learning representations of the cellular states of cells treated with different perturbagens in the LINCS project. We showed that the trained VAE and S-VQ-VAE models were able to accurately regenerate transcriptomic profiles almost indistinguishable from the input data. These results are intriguing because they suggest that the DGMs may have captured signals of cellular processes underlying the statistical structures of the data. Such capability is highly desirable as it provides a means to investigate how responses to diverse environmental changes are encoded in cellular systems as signals.

Cellular signaling systems are essentially coding machines, and training a machine capable of mimicking the behaviors of cellular signaling systems is a critical step of using contemporary artificial intelligence technologies to advance systems biology. A more intriguing future direction is to investigate whether the signals of latent variables can be mapped to the signals encoded by real biological entities like proteins or pathways as indicated by the previous research^28^. This may require further development of interpretable deep learning models that integrate information from multiple platforms. In our development of the models, we compared VAE with other DGMs, including restricted Boltzmann machines^31^, deep belief networks^31^, deep autoencoders^15^, and VQ-VAEs^18^. VAE outperformed all of these DGMs in capturing the expression data distribution. However, in its original form, VAE cannot utilize additional information aside from data passed from the input layer. The S-VQ-VAE model is an early attempt toward the goal of combining different information sources. It utilizes additional label information to facilitate the learning of global representations, but essentially it does not directly combine multiple types of data nor realize a fully interpretable multi-task learning. More directions of model design remain to be explored. We believe that additional information such as genetic perturbations can be used to determine to which biological entity the signal encoded by a latent variable likely corresponds. As cells are the basic unit of life, a complete model for understanding cellular signaling systems would represent a major breakthrough in both machine learning and systems biology, with profound implications for cell biology, pharmacology, drug development, and precision medicine.

## Methods

### Data

The SMP dataset was extracted from the Gene Expression Omnibus (GEO) dataset GSE70138^32^, which contained the level 5 L1000 expression data (moderate z-scores) of the 978 landmark genes of 85,183 samples from seven major cell lines treated with small molecules (Supplementary Table 1). The GP dataset was obtained from the GEO dataset GSE106127^33^, which contained the level 5 data of 116,782 samples from nine major cell lines with gene knockdowns (Supplementary Table 1). A cell line was considered as a major cell line if the cell line had over 10,000 samples. We performed PCA on the two datasets, and the distributions of samples in the first two principal components are shown in Supplementary Fig. 2. By comparing the scatter plot of the SMP dataset with its density contour (Supplementary Fig. 2), we can see that the group of samples on the right of the scatters plot is an outlier group with high variance but low density. This group contained 4649 samples treated with two proteasome inhibitors, bortezomib, and MG-132. Therefore, in the third dataset, the SMGP dataset that merges the SMP dataset with the GP dataset, these outlier samples were excluded. The removal of outliers resulted in comparable distributions between SMP samples and GP samples (Supplementary Fig. 2), which enabled the use of the SMGP dataset for training a VAE model to reveal connections between small molecules and knocked down genes.

The SMC dataset was a subset of the SMP dataset that contained 12,079 samples treated with 204 small molecules belonging to 75 PCLs. The PCL information was extracted from Supplementary Table 7 of the original L1000 paper^7^. The SMC dataset was used to train LR and SVM models for predicting PCLs of samples based on cellular representations learned from VAEs.

The dataset used to learn PCL representations with S-VQ-VAE was a subset of the SMC dataset, the SMCNP dataset (Supplementary Table 2), which excluded the samples treated with the proteasome inhibitor MG-132 (bortezomib was not given a PCL label, and thus had been excluded from the SMC dataset). This subset contained 9769 samples treated with small molecules from 75 PCLs.

### S-VQ-VAE model

S-VQ-VAE is a new DGM designed in this study for learning a vector representation (embedding) for each PCL. The model was extended from the standard VQ-VAE^18^ by adding a supervised mapping step to guide the training of the embedding space. Like VQ-VAE, a S-VQ-VAE is composed of three parts, an encoder neural network to generate the encoding vector $z_e(x)$ given an input vector $x$, an embedding space to look up the discrete representation $z_q(x)$ based on $z_e(x)$, and a decoder neural network to reconstruct the input data from $z_q(x)$ (Fig. 1b). Suppose that the encoder encodes the input data to a vector of length $D$, the embedding space $E$ is then defined as $E \in R^{Y \times D}$, where $Y$ is the number of different classes of the input data. In our case, this corresponds to PCLs. Each of the $Y$ embedding vectors of dimension $D$ is designated to learn a global representation of one of the classes. In forward computation, an input $x$ is first converted to its encoding vector $z_e(x)$, which will be used to update the embedding space. In the training phase, $z_e(x)$ is replaced with $z_q\left( x \right) = e_y$ to pass to the decoder, where $e_y$ is the embedding vectors of the class $y$ of $x$. In the testing phase, $z_e(x)$ is replaced by its nearest code $z_q\left( x \right) = e_k$ with

1 $$k = \mathop {\text{argmin}}\limits_j\left\| {z_e\left( x \right) - e_j} \right\|$$

Note that we are not assuming a uniform distribution of the embedding vectors as in the ordinary VQ-VAE^18^. Instead, the distribution of codes is determined by the input data with its discrete class labeling governing by a multinomial distribution.

In order to design a model that can learn individual representations through data reconstruction as well as learn a global representation for each class in a supervised manner, the objective function of S-VQ-VAE contains a reconstruction loss to optimize the encoder and decoder (first term in Eq. (2)), and a dictionary learning loss to update the embedding space (second term in Eq. (2)). The form of reconstruction loss can be selected based on the data type, and here we used the mean square error (MSE). Following the training protocol of standard VQ-VAE^18^, we chose VQ as the dictionary learning algorithm, which computes the L2 error between $z_e(x)$ and $e_y$ thus updating the embedding vector towards the encoding vector of an input case of class $y$. To control the volume of the embedding space, we also added a commitment loss between $z_e(x)$ and $e_y$ to force the individual encoding vector towards to the corresponding global embedding vector (third term in Eq. (2)).

2 $$L = l_r( {x,d( {e_y})}) + \left\| {sg\left[ {z_e\left( x \right)} \right] - e_{y}} \right\|_2^2 + \beta \left\| {z_e\left( x \right) - sg\left[ {e_y} \right]} \right\|_2^2-I\left( {k \,\ne\, y} \right)( {\left\| {sg\left[ {z_e\left( x \right)} \right] - e_k} \right\|_2^2 + \gamma \left\| {z_e\left( x \right) - sg\left[ {e_k} \right]} \right\|_2^2})$$

In addition to making the encoding vectors and the embedding vectors converge, we added two additional terms to force the encoding vector of an input data to deviate from the nearest embedding vector $e_k$ if $k\, \ne\, y$ (i.e., to minimize misclassification with the nearest neighbor). As given in Eq. (2), the fourth term is another VQ objective which updates the embedding vector of the mis-class. The final term, called the divergence loss, expands the volume of the embedding space in order to allow different classes to diverge from each other. Coefficients are applied to the commitment loss ($\beta$) and divergence loss ($\gamma$) to control the strength of regularization over the embedding space volume. According to preliminary experiments using coefficients from [0, 1], the performance of the model is quite robust to these coefficients. For generating the results presented in this study, we used $\beta = 0.25$, and $\gamma = 0.1$. Note that the mapping step with either the class label or nearest neighbor has no gradient defined for it. As in VQ-VAE, we approximate the gradient in a manner similar to the straight-through estimator^34^, by passing the gradient from the reconstruction loss from $z_q(x)$ directly to $z_e(x)$.

As a generative model, S-VQ-VAE can also be used to generate new data from the distribution of the training data. The data generation process is composed of two steps, similar to the ancestral sampling method. First, sample a target class $y$ from the distribution of classes of the input data. Second, sample an encoding vector $z\sim N(e_y,\sigma ^2)$, where $\sigma ^2$ is the covariance matrix of hidden variables estimated from the training data of class $y$. A new sample of class $y$ can then be generated by passing *z* to the decoder of S-VQ-VAE. The generation process reflects another advantage of S-VQ-VAE compared to unsupervised GMs: we can determine what content the new data should present rather than interpret it afterward.

In this study, we only utilized the global representation learning function of S-VQ-VAE. The test phase and the new data generation function of S-VQ-VAE were not examined here. To see how S-VQ-VAE can be used as a general generative model, please refer to our tutorial of S-VQ-VAE at https://github.com/evasnow1992/S-VQ-VAE, where we provide an example applying S-VQ-VAE on a benchmark machine learning dataset, the MNIST handwritten digits data^35^.

### Model architecture and training setting

The VAE model we implemented had three hidden layers in its encoder and three hidden layers in its decoder; the third hidden layer of the encoder was shared by both the encoder and decoder parts via a sampling step (Supplementary Fig. 1) and is also called the top hidden layer. The structure of the encoder was 978-1000-1000-100, where we had 978 nodes in the input layer, each corresponding to a landmark gene in the LINCS data, 1000 nodes in the first and second hidden layers, and 100 nodes in the third hidden layer (Supplementary Fig. 1). The structure of the decoder was just the reverse of the encoder. We only included the 978 landmark genes as input data to avoid redundant information from the inferred expression levels of other genes. The number of hidden layers and the number of nodes on each layer were determined based on preliminary experiments with a wide range of model architectures. Specifically, we tried architectures from 978-500-15 to 978-2000-1000-200 to select a model with as a simple structure as possible and with a low training error. Based on our previous experience, a three hidden layer model with 1000–1500 nodes on the first hidden layer, ~1000 nodes on the second hidden layer and small bottleneck on the third hidden layer usually performs the best^28,36^. The best model we achieved in this study had a structure of 978-1000-1000-100.

We used a standard normal distribution, $N(0,1)$, as the prior distribution of the top hidden layer variables $p(z)$ of VAE. The input data of our models were the L1000 level 5 gene expression data of range [−10, +10]. In order to preserve the sign information of the input data, where a positive value indicates high-expression of a gene and a negative value indicates low expression of a gene, we chose the tangent function as the activation function for all hidden layers. Note that the tangent function will map a real number to [−1, +1], while our input data are of range [−10, +10]. In order to reconstruct the input data, the outputs of the last layer of the decoder were rescaled to [−10, +10] before computing the reconstruction loss (Supplementary Fig. 1).

The loss/target function for training a general VAE is

3 $$L = l_r(x,d(z_{e\left( x \right)})) + KL(q(z|x)||p\left( z \right))$$

where the first term is the reconstruction loss and the second term is the KL-distance between the posterior distribution of the top hidden variables $q(z|x)$ given the input data and the prior variational distribution $p(z)$. In our implementation, we computed the MSE as the reconstruction loss. We trained three VAE models using the SMP, GP, and SMGP datasets independently. Each model was trained on 9/10 (random split) of the data and validated on the other 1/10 data. All models were trained for 300 epochs, with batch size 512 and learning rate 1e-3 (Supplementary Table 2). To generate a new sample, we first sampled from the multi-variate $N(0,1)$ distribution to get an encoding vector, then passed the vector through the decoder of the VAE to generate a new data.

The S-VQ-VAE model we implemented had a single hidden layer of 1000 nodes in its encoder. The decoder was the reverse of the encoder. As in VAE, we also used the tangent activation function for S-VQ-VAE and rescaled the data from [−1, 1] to [−10, 10] before computing the reconstruction loss. The number of hidden layers and hidden nodes were selected based on preliminary experiments with architectures from one to two hidden layers and 200 to 1500 hidden nodes in each layer. The embedding space contained 75 codes, one for each PCL. The model was trained on 9/10 (random split) of the SMCNP dataset for 900 epochs, with batch size 256, and learning rate 1e-4. The model was validated on the other 1/10 data (Supplementary Table 2).

### Mixing score of binary-categorical data

To quantize the mixing level of the two types of data (real expression profiles vs. generated expression profiles in our case), we defined a mixing score for a k-clustering result of binary-categorical data as follows. Suppose the total number of data to be clustered is $N$. For a cluster $i$, the number of data in this cluster of one category is denoted as $p_i$, and the number of data of the other category is denoted as $q_i$. Then the mixing score for a k-clustering result is defined as

4 $$MS_k = \frac{{\mathop {\sum }\nolimits_{i = 1}^k {\mathrm{max}}(p_i,q_i)}}{N}$$

This score equals the average proportions of data from the category that dominates each cluster. The mixing score is of range [0.5, 1], where 0.5 indicates the two categories on average mixing evenly in the $k$ clusters, and 1 indicates the two categories are cleanly separated among the $k$ clusters. The mixing score tends to increase with the number of clusters $k$ used to stratify the data.

### PCL prediction

Seven different types of sample representations were evaluated as predictors for predicting the PCL label of the small molecule that treated each SMC sample via LR, random forest, naive Bayes classifier, and SVM. We only reported the results of LR and SVM here as these two models consistently outperformed the others, and LR achieved the best validation performance while SVM was significantly more tolerant of overfitting. The seven representation types included the raw expression profile, the latent representations from three encoder layers, the 12 signature nodes values, and the latent representations from two decoder layers of the SMGP-trained VAE (the top hidden layer of the encoder is shared with the decoder, thus there are only two independent decoder layers). The latent representation of a layer of a sample was obtained by feeding the expression profile of the sample to the pre-trained VAE and extracting the values of hidden nodes on the desired layer. The prediction accuracy and Cohen’s Kappa score reported in this study were obtained by doing 10-fold cross-validation across SMC data. Specifically, the SMC data were randomly split into 10 subsets. In each iteration, an independent model was trained on 9 of the subsets and validated on the 10th subset. The reported accuracies and Kappa scores are the averages taken over the 10 models.

### Drug-target identification

The known drug-target relationships from ChEMBL database^29^ were extracted referring to Table 1 of Pabon el al.^12^, which included 16 drugs tested in all seven major cell lines in the SMP dataset. The known drug-target relationships from DGIdb^30^ were extracted from the online data portal, which included 810 drugs tested in all major cell lines in the SMP dataset. We considered different LINCS drug IDs with the same drug name as the same perturbagen.

For identifying gene targets for each drug related to the results shown in Table 1 and Supplementary Table 4, we first extracted samples treated with the drug from the SMP dataset. Then for each sample, we computed the Pearson correlations between the representation of the sample and the corresponding representations of all 116,782 samples from the GP dataset. The genes knocked down in the GP samples were ranked according to the Pearson correlations, and the rank of the top known target gene was recorded. Finally, the best top rank and mean top rank across all samples treated with the same drug were computed and used to compare different types of representations. Similar to PCL classification, seven types of sample representations were compared based on the top rank and mean rank.

When computing the aggregation performance for Supplementary Table 5, we only included SMP and GP samples that were perturbed with drugs or their target gene knockdowns from the known drug-target relationships. Bortezomib from ChEMBL was excluded from this experiment as SMP samples perturbed by this drug (a proteasome inhibitor) are not distribution consistent with other samples as indicated before.

### Program language, packages, and softwares

VAE and S-VQ-VAE models were implemented in Python2.7 using the library PyTorch 0.4.1^37^. Adam optimizer was used for updating the models. PCA analysis, LR functions, and SVM functions were from the Python library Scikit-learn 0.21.3^38^. For LR, we used random seed 0 for shuffling data and solver “lbfgs” (Limited-memory BFGS) for multi-classification. For SVM we used random seed 0 and default settings for the other hyper-parameters. Distance computation functions, including Euclidean distance and Pearson correlation, related to Figs. 3b, 4, 5, and drug-target prediction were from the Python library SciPy 1.3.1^39^. For Figs. 3b and 4 we used the Euclidean distance for revealing general associations between expression profile representations and for Fig. 5 and drug-target prediction we used the Pearson correlation for emphasizing more on the orientation consistency between representations. Hierarchical clustering and heatmap visualization related to Fig. 3 were carried out with the Python library Seaborn 0.9.0^40^. The code for preprocessing LINCS data, training VAE and S-VQ-VAE models, and carrying out model analyses are available at https://github.com/evasnow1992/DeepGenerativeModelLINCS. S-VQ-VAE PCL representation graph visualization and community detection related to Fig. 5 were accomplished with software Gephi 0.9.2^41^. The community detection algorithm being used was the Louvain algorithm developed by Blondel et al.^22^ and was run with randomization (for better decomposition), using edge weights, and resolution 1 (for detecting smaller communities).

### Reporting summary

Further information on experimental design is available in the Nature Research Reporting Summary linked to this paper.

## Supplementary information

Supplementary information

Reporting Summary Checklist

## Data Availability

The LINCS L1000 datasets analyzed during the current study are available in the GEO repository with accession codes GSE70138 and GSE106127. The DGIdb drug-gene interaction dataset that supports the findings of this study can be downloaded from the online data portal (http://www.dgidb.org/downloads). No datasets were generated during the current study.

## Code availability

The code generated during the current study and the pretrained models have been deposited to GitHub at https://github.com/evasnow1992/DeepGenerativeModelLINCS with the MIT license. An independent tutorial of applying the S-VQ-VAE model on the benchmark MNIST dataset is available at https://github.com/evasnow1992/S-VQ-VAE. Instructions for running the code and intermediate results for an example run are presented along with the code.
