# Supplementary material for: Learning to encode cellular responses to systematic perturbations with deep generative models
Source: NPJ Syst Biol Appl. 2020 Nov 6;6:35. doi: 10.1038/s41540-020-00158-2 (PMC7648057; doi:10.1038/s41540-020-00158-2)
Supplement: Supplementary file 1 — Supplementary information [file 41540_2020_158_MOESM1_ESM.pdf]

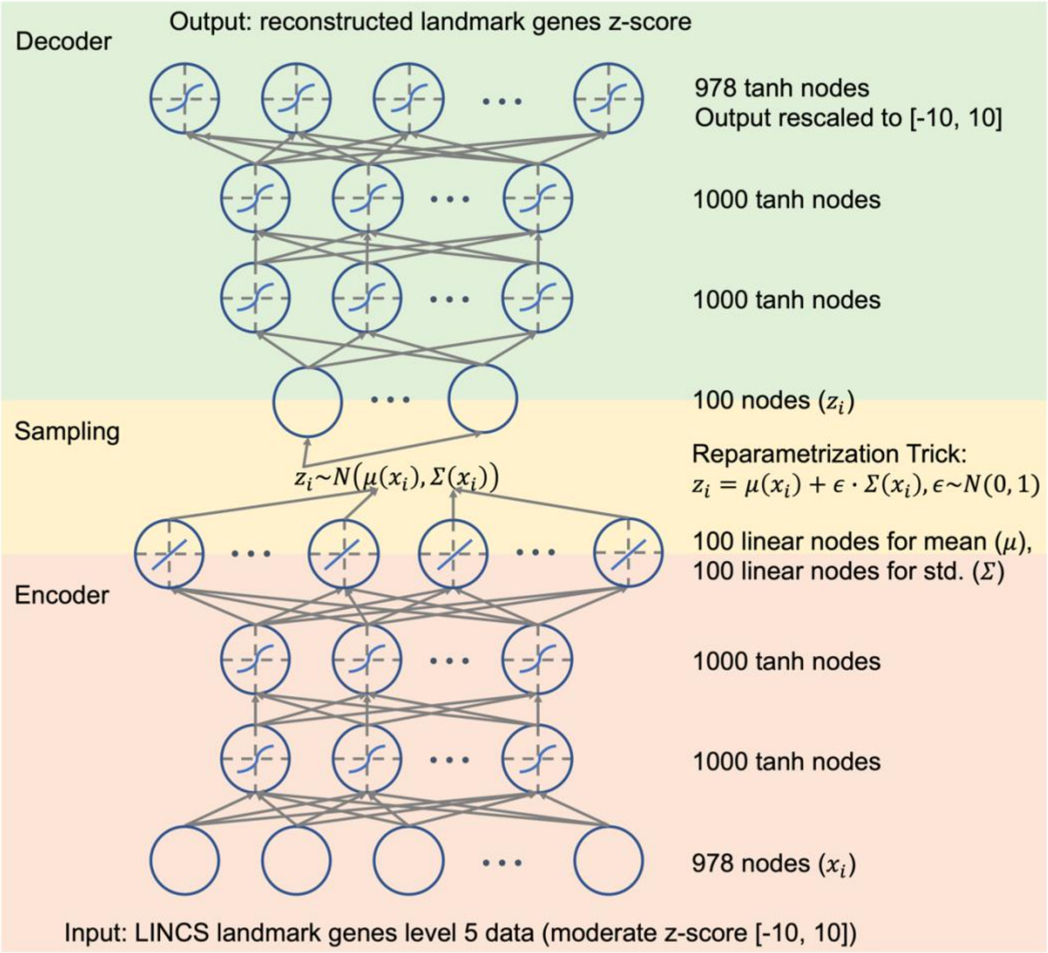

Supplementary Figure 1. The architecture of VAE model. The encoder is composed of three hidden layers, where the third hidden layer defines the distribution from which the encoding vector z_i is sampled. The reparameterization trick is applied to generate a differentiable estimate of z_i, which allows gradient descent to be used for training the model. The decoder has a reversed architecture as the encoder.

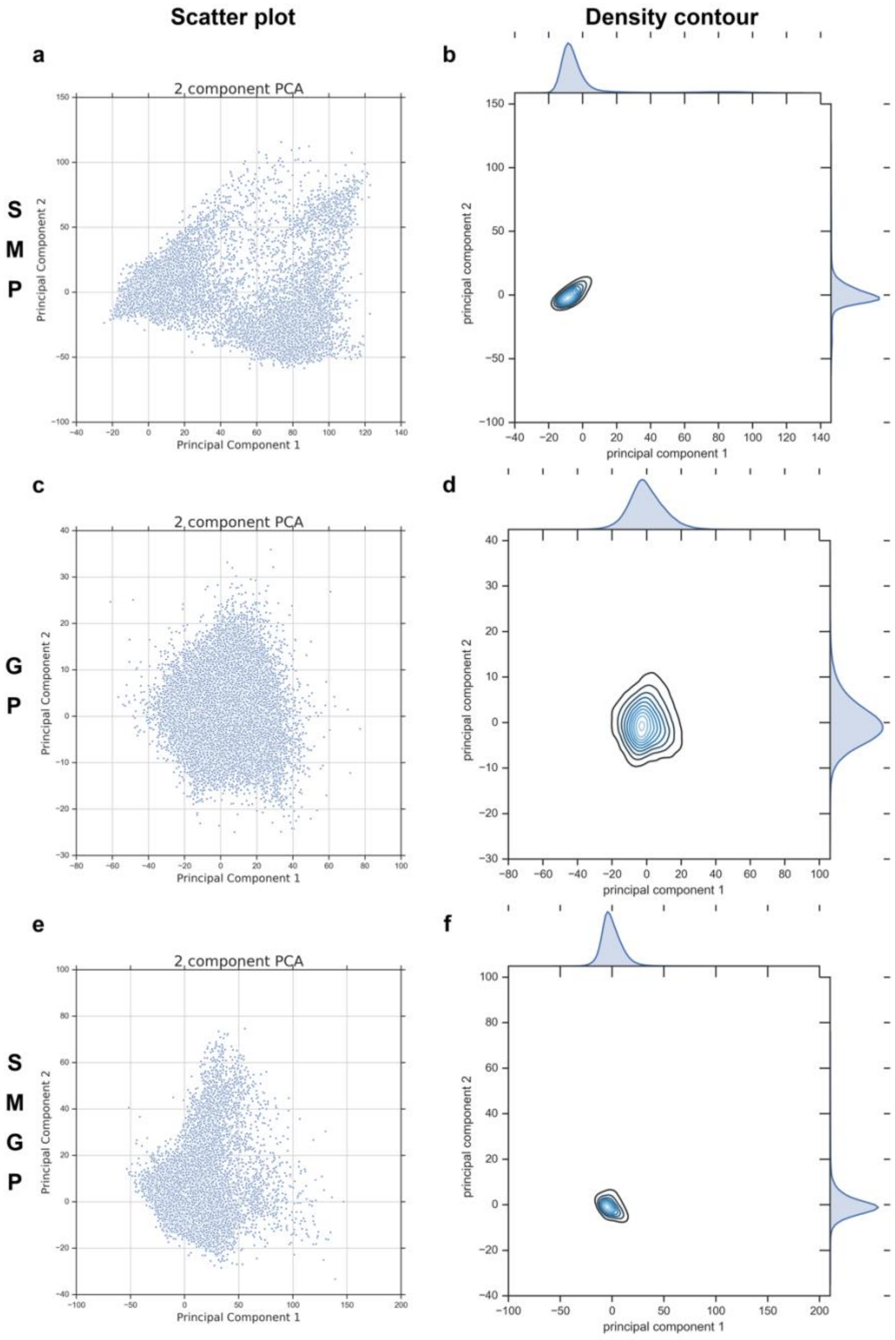

Supplementary Figure 2. PCA two components scatter plots and density contour plots of three input datasets. (a and b) Scatter plot and density contour of the SMP dataset. The outlier group on the right of the scatter plot is composed of 4,649 samples treated with bortezomib and MG-132. Both these SMPs are proteasome inhibitors. (c and d) The scatter plot and density contour of the GP dataset. (e and f) The scatter plot and density contour of a combination of the SMP and GP datasets (the SMGP dataset), excluding the outlier group of proteasome inhibitors.

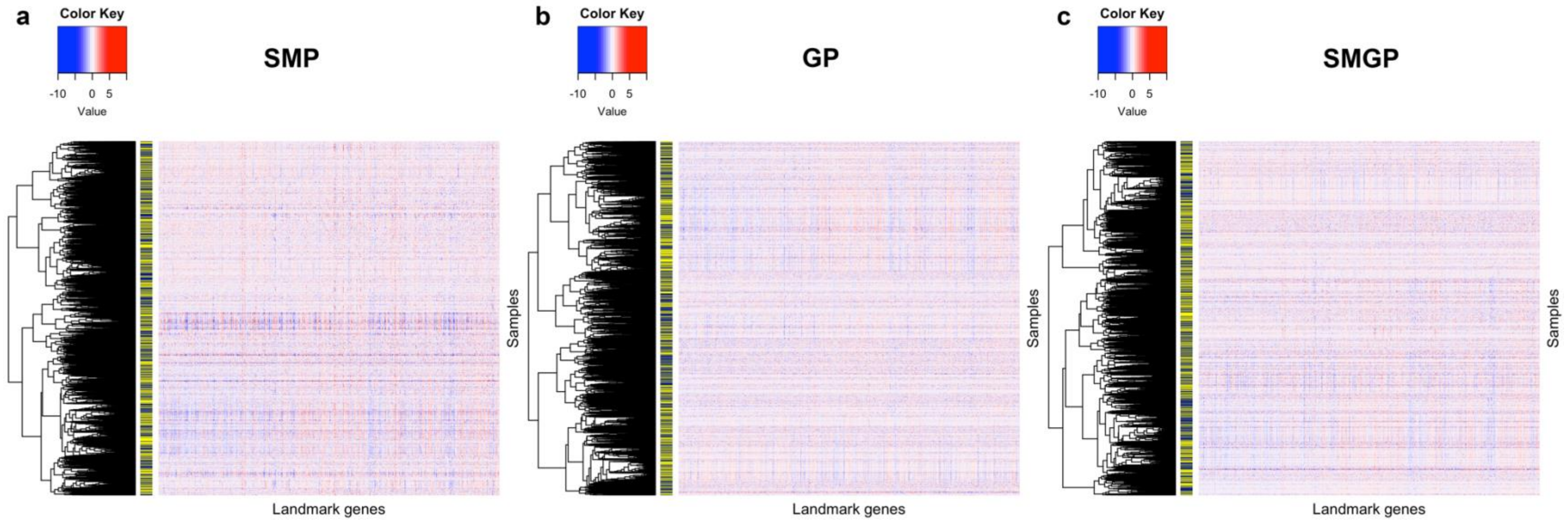

Supplementary Figure 3. Hierarchical clustering of simulated data generated by trained VAEs vs. real data from the corresponding training datasets. (a-c) The plot of 2,000 data generated with SMP-trained VAE, GP-trained VAE, and SMGP-trained VAE, respectively, and 2,000 real data randomly selected from the corresponding training datasets. In the row color bar of the heatmap, blue indicates the original data and yellow indicates the simulated data. The evenly mixing of the original data and simulated data suggests that the simulated data are indistinguishable from the original data by doing a hierarchical clustering using 1-Pearson Correlation as distance.

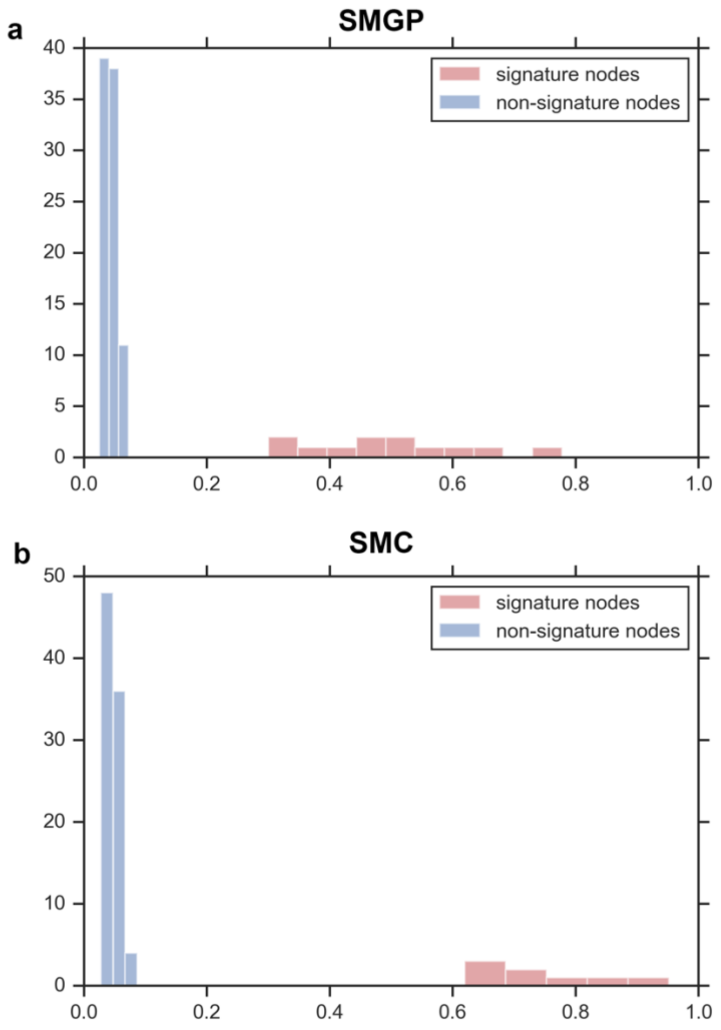

Supplementary Figure 4. Histograms of average absolute node value of 12 signature nodes and 88 non-signature nodes on the top hidden layer of SMGP-trained VAE. (a) The average was taken over all SMGP samples. (b) The average was taken over all SMC samples.

Supplementary Table 1. LINCS datasets and major cell lines.

GEO ID	Dataset content	# of sample	# of perturbagen	# of drug/gene name	# of perturbagen class (PCL)	# of cell line	Major cell line[1] name	Major cell line[1] type	# of sample in cell line
GSE70138	LINCS phase II L1000 dataset, mainly small molecular perturbation	118050	2170	1826	991 perturbagens in 171 classes	41	MCF7	breast adenocarcinoma	13476
							A375	malignant melanoma	12740
							PC3	prostate adenocarcinoma	12719
							HT29	colorectal carcinoma	12529
							HA1E	normal kidney	12481
							YAPC	pancreatic carcinoma	10621
							HELA	large intestine adenocarcinoma	10617
GSE106127	LINCS L1000 RNAi and CRISPR dataset. Corresponds to genetic perturbational signatures of shRNAs and CRISPR reagents, that exist in GEO series GSE70138 and GSE92742.	119013	18413	4320	NA	15	VCAP	prostate carcinoma	17098
							A375	malignant melanoma	13121
							PC3	prostate adenocarcinoma	13061
							HA1E	normal kidney	12957
							A549	non-small cell lung cancer	12691
							HT29	colorectal carcinoma	12305
							HCC515	lung cancer	11985
							MCF7	breast adenocarcinoma	11869
							HEPG2	hepatocellular carcinoma	11695

[1]A major cell line is a cell line that has over 10,000 samples.

Supplementary Table 2. The data reconstruction performance of VAE models and S-VQ-VAE model on training data and validation data.

Model type	Train data	Training loss[1]	Validation loss[1]
VAE	SMP	1.081	1.113
VAE	GP	0.861	0.864
VAE	SMGP	0.995	1.002
S-VQ-VAE	SMCNP	1.725	1.831

[1]The models were trained on 9/10 of the data and validated on the other 1/10 of the data. The reported losses are MSE between the reconstructed data and the input data.

Supplementary Table 3. Performance of PCL classification with different sample representations as input data.

Representation type	LR[1] test accuracy[2]	LR test Cohen's Kappa score	SVM[1] test accuracy	SVM test Cohen's Kappa score
raw	0.5922	0.5675	0.5273	0.4989
encoder layer 1	0.5096	0.4794	0.4528	0.4199
encoder layer 2	0.4461	0.4131	0.3881	0.3540
encoder layer 3	0.4098	0.3704	0.4232	0.3851
signature nodes	0.3814	0.3399	0.3615	0.3168
decoder layer 1	0.4002	0.3599	0.4082	0.3683
decoder layer 2	0.3994	0.3573	0.4085	0.3684

[1]LR: logistic regression; SVM: support vector machine.

[2]The accuracy and Cohen's Kappa score were obtained with 10-fold cross validation.

Supplementary Table 4. The mean of rank of the top known target for 16 FDA-approved drugs from drug-target prediction with different types of representation.

Drug	Target	# of SMP[1]	# of GP	Raw	E1	E2	T	Sig.	D1	D2
pitavastatin	HMGCR	42	45	**376.40**[2]	962.69	1930.62	806.81	706.74	827.60	688.00
bortezomib	PSMB10, PSMA3, PSMA1, PSMA5, PSMB7, PSMB5, PSMA8, PSMB1	2339	196	20.32	48.12	45.94	65.69	96.78	49.09	**19.13**
hydrocortisone	NR3C1	42	36	4605.67	3759.6	2221.74	3019.07	3339.52	**2122.33**	2392.12
vemurafenib	BRAF	81	108	1030.01	981.65	1215.56	779.80	**664.57**	970.10	1060.69
flutamide	AR	42	29	**5111.36**	7178.43	8167.55	9172.00	11013.07	13934.31	13047.52
clobetasol	NR3C1	42	36	5221.50	5025.12	3236.19	5060.95	5628.48	**3136.02**	4185.90
digoxin	ATP1A3, FXYD2, ATP1B1	42	83	595.62	431.10	492.21	497.86	**336.48**	590.71	405.69
mycophenolate-mofetil	IMPDH2	42	27	**4594.12**	6514.81	5928.67	6630.00	5091.55	8631.02	4683.26
dasatinib	LCK, YES1	204	175	942.70	743.19	694.73	681.38	**641.82**	649.23	658.83
amlodipine	CACNA1D	42	27	7798.79	4797.38	5290.60	5252.71	5159.55	5872.90	**4295.33**
calcitriol	VDR	42	27	5293.00	5859.21	6924.50	5877.14	6128.60	**5263.52**	5856.98
glibenclamide	KCNJ11	42	27	6074.93	9643.98	8653.05	7038.45	**3969.43**	7179.50	7104.81
paclitaxel	TUBB6, TUBA1A, TUBB2A, TUBB2C	42	105	464.17	548.00	781.81	640.90	**412.62**	421.00	831.40
losartan	AGTR1	42	24	4841.31	4767.88	4162.24	3865.98	3871.10	**3656.90**	4469.76
irinotecan	TOP1	42	27	5079.93	5317.57	4332.83	4459.55	4248.19	3999.52	**3915.17**
raloxifene	ESR2	42	36	4686.60	3236.07	**1262.83**	2078.57	2383.14	1595.02	1757.98

[1]# of SMP: the number of SMP samples treated with the drug; # of GP: the number of GP samples with target gene knocked down; E: encoder layer; T: top hidden layer; Sig.: signature nodes; D: decoder layer.

[2]Data related to Figure. 6. The value for a given drug and a type of representation is the mean rank of the drug target gene(s) among all genes in GP dataset when retrieved and ranked according to the similarity between the representations of SMP samples treated with the drug and representations of all GP samples. The lowest mean rank is bolded for each drug.

Supplementary Table 5. Aggregation drug target identification performance for drugs from ChEMBL and DGIdb.

Database		ChEMBL[2]	DGIdb[3]
# of drugs		15	810
# of target genes		28	816
# of SMP[1]		831	37,015
# of GP		972	26,262
Average recall at 10/at 100	Raw	0.305	0.147
	E1	0.334	0.147
	E2	0.366	0.147
	T	0.381	0.144
	Sig.	0.405	0.145
	D1	0.381	0.145
	D2	0.361	0.054
Prop. of drugs with at least one target gene among top 10/100	Raw	0.403	0.313
	E1	0.442	0.317
	E2	0.471	0.316
	T	0.486	0.305
	Sig.	0.521	0.308
	D1	0.483	0.308
	D2	0.472	0.146

[1]# of SMP: the total number of SMP samples treated with the drugs; # of GP: the total number of GP samples with target gene knocked downs; E: encoder layer; T: top hidden layer; Sig.: signature nodes; D: decoder layer.

[2]For ChEMBL, measurements were computed based on top 10 retrieval. Bortezomib from ChEMBL was excluded as proteasome inhibitors can lead to sample distribution inconsistency.

[3]For DGIdb, measurements were computed based on top 100 retrieval.
