# Supplementary material for: Learning to encode cellular responses to systematic perturbations with deep generative models
Source: NPJ Syst Biol Appl. 2020 Nov 6;6:35. doi: 10.1038/s41540-020-00158-2 (PMC7648057; doi:10.1038/s41540-020-00158-2)
Supplement: Supplementary file 2 — Reporting Summary Checklist [file 41540_2020_158_MOESM2_ESM.pdf]

nature research

Corresponding author(s): Xinghua Lu

Last updated by author(s): Jul 29, 2020

Reporting Summary

Nature Research wishes to improve the reproducibility of the work that we publish. This form provides structure for consistency and transparency in reporting. For further information on Nature Research policies, see Authors & Referees and the Editorial Policy Checklist.

Statistics

For all statistical analyses, confirm that the following items are present in the figure legend, table legend, main text, or Methods section.

n/a	Confirmed	
☐	☒	The exact sample size (*n*) for each experimental group/condition, given as a discrete number and unit of measurement
☒	☐	A statement on whether measurements were taken from distinct samples or whether the same sample was measured repeatedly
☐	☒	The statistical test(s) used AND whether they are one- or two-sided<br>*Only common tests should be described solely by name; describe more complex techniques in the Methods section.*
☐	☒	A description of all covariates tested
☐	☒	A description of any assumptions or corrections, such as tests of normality and adjustment for multiple comparisons
☐	☒	A full description of the statistical parameters including central tendency (e.g. means) or other basic estimates (e.g. regression coefficient) AND variation (e.g. standard deviation) or associated estimates of uncertainty (e.g. confidence intervals)
☐	☒	For null hypothesis testing, the test statistic (e.g. *F*, *t*, *r*) with confidence intervals, effect sizes, degrees of freedom and *P* value noted<br>*Give P values as exact values whenever suitable.*
☐	☒	For Bayesian analysis, information on the choice of priors and Markov chain Monte Carlo settings
☐	☒	For hierarchical and complex designs, identification of the appropriate level for tests and full reporting of outcomes
☒	☐	Estimates of effect sizes (e.g. Cohen's *d*, Pearson's *r*), indicating how they were calculated

Our web collection on statistics for biologists contains articles on many of the points above.

Software and code

Policy information about availability of computer code

Data collection	No software was used for data collection
Data analysis	Custom Python code for constructing the models and analyzing the data have been deposited to GitHub at https://github.com/evasnow1992/DeepGenerativeModelLINCS. Software Gephi 0.9.2 was used for graph visualization and community detection related to Figure 5.

For manuscripts utilizing custom algorithms or software that are central to the research but not yet described in published literature, software must be made available to editors/reviewers. We strongly encourage code deposition in a community repository (e.g. GitHub). See the Nature Research guidelines for submitting code & software for further information.

Data

Policy information about availability of data

All manuscripts must include a data availability statement. This statement should provide the following information, where applicable:

- Accession codes, unique identifiers, or web links for publicly available datasets
- A list of figures that have associated raw data
- A description of any restrictions on data availability

The datasets analyzed during the current study are available in the GEO repository with accession codes GSE70138 and GSE106127 (https://www.ncbi.nlm.nih.gov/geo/query/acc.cgi?acc=GSE70138; https://www.ncbi.nlm.nih.gov/geo/query/acc.cgi?acc=GSE106127).

Field-specific reporting

Please select the one below that is the best fit for your research. If you are not sure, read the appropriate sections before making your selection.

☒ Life sciences ☐ Behavioural & social sciences ☐ Ecological, evolutionary & environmental sciences

For a reference copy of the document with all sections, see nature.com/documents/nr-reporting-summary-flat.pdf

Life sciences study design

All studies must disclose on these points even when the disclosure is negative.

Sample size	No sample-size calculation was performed.
Data exclusions	4,649 samples treated with two proteasome inhibitors, bortezomib, and MG-132, were excluded from training models as they formed an outlier distribution when comparing to the other 197,316 samples on a principal component analysis plot. More details are provided in the Data subsection in Methods.
Replication	All experiments were first carried out on subsets of data from a single cell line, e.g. A375 and MCF7, before transferring to the data combining all available cell lines. All major conclusions about the data distribution, model properties, signature nodes, and following supervised learning performance were consistent between a using a subset of data and all data. Different random split of data into training and validation datasets were also compared. All attempts at replication were successful.
Randomization	All datasets were randomly split into a training dataset containing 90% of the data and a validation dataset containing 10% of the data. 10-fold cross validation was carried out for PCL prediction.
Blinding	The investigator were blinded to data collection and dataset split process.

Reporting for specific materials, systems and methods

We require information from authors about some types of materials, experimental systems and methods used in many studies. Here, indicate whether each material, system or method listed is relevant to your study. If you are not sure if a list item applies to your research, read the appropriate section before selecting a response.

Materials & experimental systems

n/a	Involved in the study	
☒	☐	Antibodies
☒	☐	Eukaryotic cell lines
☒	☐	Palaeontology
☒	☐	Animals and other organisms
☒	☐	Human research participants
☒	☐	Clinical data

Methods

n/a	Involved in the study	
☒	☐	ChIP-seq
☒	☐	Flow cytometry
☒	☐	MRI-based neuroimaging
